# Mild steel and stainless steel welding fumes elicit pro‐inflammatory and pro‐oxidant effects in first trimester trophoblast cells

**DOI:** 10.1111/aji.13221

**Published:** 2020-02-03

**Authors:** Nicole S. Olgun, Anna M. Morris, Lauren N. Bowers, Aleksandr B. Stefaniak, Sherri A. Friend, Sandra E. Reznik, Stephen S. Leonard

**Affiliations:** ^1^ Health Effects Laboratory Division National Institute for Occupational Safety and Health Morgantown West Virginia; ^2^ Respiratory Health Division National Institute for Occupational Safety and Health Morgantown West Virginia; ^3^ Department of Pharmaceutical Sciences St. John's University Queens New York

**Keywords:** inflammation, metals, trophoblast, welding

## Abstract

**Problem:**

As more women join the skilled‐trade workforce, the effects of workplace exposures on pregnancy need to be explored. This study aims to identify the effects of mild steel and stainless steel welding fume exposures on cultured placental trophoblast cells.

**Method of study:**

Welding fumes (mild steel and stainless steel) were generously donated by Lincoln Electric. Electron microscopy was used to characterize welding fume particle size and the ability of particles to enter extravillous trophoblast cells (HTR‐8/SVneo). Cellular viability, free radical production, cytokine production, and ability of cells to maintain invasive properties were analyzed, respectively, by WST‐1, electron paramagnetic resonance, DCFH‐DA, V‐plex MULTI‐SPOT assay system, and a matrix gel invasion assay.

**Results:**

For all three welding fume types, average particle size was <210 nm. HTR‐8/SVneo cells internalized welding particles, and nuclear condensation was observed. Cellular viability was significantly decreased at the high dose of 100 µg/mL for all three welding fumes, and stainless steel generated the greatest production of the hydroxyl radical, and intracellular reactive oxygen species. Production of the cytokines IL‐1β and TNFα were not observed in response to welding fume exposure, but IL‐6 and IL‐8 were. Finally, the invasive capability of cells was decreased upon exposure to both mild steel and stainless steel welding fumes.

**Conclusion:**

Welding fumes are cytotoxic to extravillous trophoblasts, as is evident by the production of free radicals, pro‐inflammatory cytokines, and the observed decrease in invasive capabilities.

## 1 INTRODUCTION

The United States is in the midst of a skilled‐trade worker shortage, which includes welders.^1^, ^2^ In a field traditionally dominated by men, the American Welding Society (AWS) estimates that nearly 300 000 welding positions will need to be filled by 2020 due to retirements, with women expected to fill many of these roles.^3^, ^4^, ^5^ According to the Department of Labor Women's Bureau, women currently make up 5.5% of the welding, soldering, and brazing workforce in the United States.^6^ As more households depend on mothers to be the sole or primary breadwinner compared to 50 years ago, there is a need to understand how gestational outcomes are impacted by workplace exposures.^7^ Currently, no data are available pertaining to how many pregnant women welders are employed in the United States workforce.

The process of welding involves the joining of metals, to form a single, continuous piece.^8^ The heating of these metals generates a complex mixture of gaseous and aerosol by‐products, composed mainly of ultrafine metals, metal oxides, silicates, and fluorides.^9^ Gas metal arc welding (GMAW) and shielded metal arc welding (SMAW) are commonly used to weld mild steel (MS) and stainless steel (SS).^8^, ^10^ Fumes generated from MS are mainly composed of iron (Fe) and manganese (Mn), while SS welding fumes also contain chromium (Cr) and nickel (Ni).

In studies done in both humans and the animal model, exposure to welding fumes has been associated with bronchitis, immunosuppression, metal fume fever, neurological effects, and dysfunction of the male reproductive system.^11^, ^12^, ^13^, ^14^ In 2015, as part of the Women's Health in Apprenticeship Trades‐Metalworkers and Electricians (WHAT‐ME) study, it was reported that the urinary concentrations of manganese (96%) and chromium (2%) were higher in female welders when compared to the general population, likely due to workplace exposures.^15^ Emerging evidence also suggests that heavy metals act as endocrine‐disrupting chemicals in pregnant women, by blocking, mimicking, or altering the activity of hormones and disrupting the normal growth and development of the fetus.^16^, ^17^, ^18^

With an abundance of information available on the health effects of welding fume exposure on various organ systems, and the effects of individual metals on pregnancy, there is a noticeable gap in the scientific literature concerning welding fume exposure in pregnant women. Using a human, villous‐derived trophoblast cell line from the first trimester (HTR‐8/SVneo), which is frequently used to investigate cytotoxic placental environments, this study aims to bridge that gap in knowledge.^19^, ^20^, ^21^ Though trace levels of metals such as Cr and Mn are important for biological functions, exposure to elevated concentrations of these metals and others can negatively impact maternal and fetal health.

We hypothesized that exposure of the HTR‐8/SVneo cell line to both solubilized and non‐solubilized welding fume particles would cause an increase in reactive oxygen species production and pro‐inflammatory cytokine production, and alter the normal, invasive properties of cells. Our findings will further contribute toward the understanding of how the metals found in welding fumes affect the placenta and will provide valuable insight to this understudied, potential health issue.

## 2 METHODS

### 2.1 Welding fume characterization and composition

Three welding fume samples were generously donated by Lincoln Electric. All fume collection performed was consistent with the F1.2. methodology listed by the American Welding Society (https://pubs.aws.org/Download_PDFS/F1.2-2013PV.pdf). At least 2 g of welding fume particulate was provided and run with 95% argon (Ar) and 5% carbon dioxide (CO_2_) gas shielding (with the exception of SMAW‐SS), using the following electrodes: gas metal arc welding using a MS ER70S‐3 electrode (GMAW‐MS), gas metal arc welding using a ER308L Si SS electrode (GMAW‐SS), and shielded metal arc welding using an Excalibur 307L‐17 SS electrode (SMAW‐SS).

In order to determine the amount of Cr(VI) present, Lincoln Electric performed ion chromatography within eight days of sample collection due to the tendency of Cr(VI) to reduce to Cr(III) over time, which is consistent with OSHA method ID‐215.^22^ The detection of all other elements was also determined by Lincoln Electric using X‐ray fluorescence.

### 2.2 Welding fume particle size

The distribution of welding fume particle sizes in suspension (50% dispersion medium [DM], 50% ultra‐pure sterile water) was measured using Nanosight NS300 Nanoparticle Tracking Analysis software. For analysis, a syringe pump was used to inject all samples through a low volume flow cell top plate at a constant rate at room temperature. Camera and threshold settings in the NTA instrument varied slightly between samples to ensure accurate particle sizing. Each sample was captured five times for 60 seconds, for a total capture time of 5 minutes per sample. The DM was prepared as described by Porter et al^23^ and consisted of a final concentration of 0.6 mg/mL mouse serum albumin (Millipore Sigma) and 0.1% v/v 1,2‐Dipalmitoyl‐*sn*‐glycero‐3‐phosphocholine (DPPC) (Millipore Sigma) in phosphate‐buffered saline (PBS), pH 7.4. Glucose was not added to the DM since RPMI medium already contains 2 g/L glucose.

### 2.3 Welding fume preparation

In order to separate the solubilized fraction from the non‐solubilized fraction, welding fumes were prepared at 1 mg/mL stock solutions in DM and incubated for 24 hours at 37°C in a shaking water bath. DM was used since the high salt concentration of phosphate‐buffered saline has been shown to enhance welding fume particle agglomeration, as does the presence of fetal bovine serum and other proteins normally found in culture medium.^13^, ^23^ The following morning, welding fume samples were centrifuged at 12 000 *g* for 30 minutes. The supernatants (soluble fraction) were carefully recovered and filtered using a 0.22 µm polyethersulfone membrane filter (Millipore Corp.) so as not to disturb the welding fume pellets at the bottom of the tube (non‐soluble fraction). This method has been used by other investigators.^24^, ^25^ The pellets were rinsed, weighed, and re‐suspended in dispersion medium to obtain 1 mg/mL solutions. In order to determine the amount of metals present in the soluble fraction, the United States Environmental Protection Agency Method 200.7, version 4.4, was performed, using inductively coupled plasma‐optical emission spectroscopy (ICP‐OES) instrumentation.^26^

### 2.4 Endotoxin analysis

In order to test for the presence of gram‐negative bacterial endotoxin on all the welding fume samples, the endpoint chromogenic limulus amebocyte lysate (LAL) assay was used (Lonza). Each welding fume was mixed with LAL and incubated for 10 minutes at 37°C. At the end of the incubation, a chromogenic substrate was added, and samples were incubated for an additional 6 minutes. An acidic “stop” solution was added, and the absorbance was spectrophotometrically determined at 405 nm. Absorbance was directly proportional to the amount of endotoxin present. The standard curve reflected endotoxin unit/mL (EU/mL) using *E coli* 0111:B4 endotoxin.

### 2.5 Cell culture

The HTR‐8/SVneo cell line (ATCC) is often used to study placental function since the cell population consists of normal trophoblasts, and not placental choriocarcinoma cells. Cells were cultured in RPMI‐1640 medium supplemented with 10% fetal bovine serum and 50 mg/mL of penicillin/streptomycin (Invitrogen Life Sciences). Cells were maintained at 37°C in a 5% CO_2_ in air incubator and passaged using 0.25% trypsin/0.53 mmol/L EDTA (Sigma‐Aldrich). Four independent experiments were performed with three replicates of each treatment in each experiment, and assays were performed in duplicate. Values from each experiment were averaged resulting in a final sample size of n = 4 for each condition.

### 2.6 Scanning electron microscopy

Particles were diluted 1:100 in filtered distilled water. An aliquot of 0.5 mL was vacuum‐filtered onto a 0.2 µm polycarbonate filter, and the filter was affixed onto a 13‐mm aluminum stub mount using double stick carbon tape. The mounted filter was then sputter‐coated with gold‐palladium for 2 minutes. The particles were imaged using a Hitachi S4800 field‐emission scanning electron microscope at 5 kV.

### 2.7 Transmission electron microscopy

Suspended, fixed cells were pelleted and embedded in 4% agarose. The cells were then post‐fixed with osmium tetroxide followed by en‐bloc staining with 1% tannic acid and 0.5% uranyl acetate. A graded series (50%, 70%, 90%, and 100%) of alcohols were used for dehydration. Propylene oxide served as an infiltrating agent before embedding the cells in epoxy resin and polymerizing in a 60°C oven for 48 hours. The resulting blocks were cut with a Leica EM UC7 Ultramicrotome at 70 nm thickness. The sections were placed on 200 mesh copper grids and stained with 4% uranyl acetate and Reynold's lead citrate. The samples were imaged using a JEOL 1400 transmission electron microscope.

### 2.8 Cellular viability

The water‐soluble tetrazolium (WST‐1) assay was used to determine the effect(s) of welding fume exposure on the viability of cells (Millipore Sigma). Cells were seeded at a density of 6 × 10^4^ cells in 96‐well plates. After a 24‐hour growth period, cells were incubated with welding fume at final concentrations of 10 and 100 µg/mL for either 4 or 24 hours. For the positive control, a 10 mmol/L Cr(VI) solution was used.

### 2.9 Electron paramagnetic resonance

Electron paramagnetic resonance (EPR) trapping with 5′5‐dimethylpyrroline N‐oxide (DMPO) was used to detect the presence of short‐lived free radicals. The ability of MS and SS welding fumes to produce the hydroxyl radical (^•^OH) under Fenton‐like reaction conditions in cultured cells was determined using a quartz flat cell assembly and Brüker EMX spectrometer. HTR‐8/SVneo cells were used at a final concentration of 2 × 10^6^ cells/mL, along with 250 µg/mL welding fume, and 100 mmol/L DMPO, which were mixed with sterile PBS and incubated at 37°C for 5 minutes before being loaded into the flat cell for analysis. Peak heights were representative of relative levels of spin‐trapped ^•^OH radicals. A 1 mmol/L solution of Cr(VI) was used for the positive control.

### 2.10 Intracellular ROS

Cells were seeded at a density of 6 × 10^4^ cells/well in 96‐well plates and incubated with 2′,7′‐dichlorohydrofluorescin diacetate (DCFH‐DA), a cell permeable fluoroprobe, at a final concentration of 1 mmol/L in serum‐free medium for 45 minures at 37°C. Cells were washed twice in sterile PBS, and medium was subsequently added back into the wells along with 10 or 50 µg/mL of welding fumes, or 1 mmol/L Cr(VI) as a positive control. Cells were then incubated for 0.5, 2, 4, and 6 hours at 37°C. Plates were read at 485 nm excitation/520 nm emission at the end of each respective time point to measure ROS production. For negative controls, medium and welding fume were plated in wells in the absence of DCFH‐DA, and readings were subtracted from those taken when exposed cells were present to account for any autofluorescence. For the positive control, a 10 mmol/L Cr(VI) solution was used.

### 2.11 Cytokine production

A V‐plex, pro‐inflammatory MULTI‐SPOT assay system (Meso Scale Deliveries, LLC) was used to measure the production of IL‐1β, TNFα, IL‐6, and IL‐8 in culture supernatants. Samples were processed according to the manufacturer's instructions, and results were quantified using electrochemiluminescence with a MESO QuickPlex SQ120 instrument. For the positive control, a 10 mmol/L Cr(VI) solution was used.

### 2.12 Invasion assay

A CytoSelect™ 24‐well kit was used (Cell Biolabs, Inc) for invasion assays. Cells were plated at a concentration of 7.0 × 10^5^ cells/mL in serum‐free medium on inserts coated with a uniform layer of basement membrane matrix solution, and welding fume at a final concentration of 50 µg/mL was added to inserts for 24 hours. Medium containing 10% fetal bovine serum was used in the lower chamber of the invasion plate to serve as the chemo‐attractant. After a period of 24 hours, invaded cells were stained and quantified using an Olympus IX 70 inverted microscope (Olympus) and Simple PCI software was used to obtain images. Inserts were then treated with an extraction solution and allowed to incubate for 10 minutes at room temperature on an orbital shaker. From each sample, 100 µL was then transferred to a 96‐well plate and the OD was measured at 560 nm. A 10 mmol/L solution of Cr(VI) was used for the positive control.

### 2.13 Statistical analysis

Statistical analyses of results were run using either one‐way or two‐way analysis of variance (ANOVA) models using Tukey's post hoc comparisons on GraphPad Prism version 8.02. Differences were regarded as significant when *P* < .05.

## 3 RESULTS

### 3.1 Welding fume particle characterization: elemental analysis, solubility, and sizing

**Figure 1:**
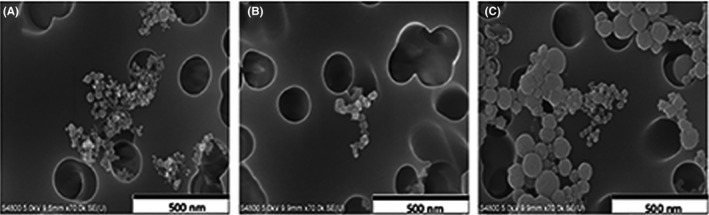
Electron microscopy of welding fume particles. Representative images using field‐emission scanning electron microscopy (FE‐SEM) are shown for (A) GMAW‐MS, (B) GMAW‐SS, and (C) SMAW‐SS. Images were acquired using 20 000× magnification using a 5.0 kV accelerating voltage

The elements present in GMAW‐MS, GMAW‐SS, and SMAW‐SS are shown in Table 1. Fe and Mn were the predominant metals found in GMAW‐MS, whereas GMAW‐SS and SMAW‐SS also contained Ni and Cr The presence of Cr in GMAW‐MS was negligible. Table 2 shows the parts per million (ppm) of solubilized metals found in welding fume preparations that were used for all studies. It was determined that Mn was the most soluble metal present in GMAW‐MS and GMAW‐SS, whereas Cr was the predominant soluble metal found in SMAW‐SS. Average particle size (Table 3) for all three welding fume types was <210 nm. Figure 1 depicts representative scanning electron micrographs for GMAW‐MS, GMAW‐SS, and SMAW‐SS. Chain‐like agglomerates of welding fume particles can be seen, and all fume types appear to be spherical and homogenous in shape.

**Table 1:** Welding fume characterization. Collection of welding fume was performed by Lincoln Electric and consistent with the F1.2. methodology listed by the American Welding Society. In order to determine the amount of Cr(VI) present, ion chromatography was performed no more than eight days from the time of sample collection since Cr(VI) reduces to CR(III) over time. This is consistent with the OSHA method ID‐215

Welding fume	Shielding gas	Element	% Detected
GMAW‐MS	95% Ar, 5% CO_2_	Fe	54.76
		Mn	6.81
		Si	1.48
		Cr(VI)	<5 ppm
GMAW‐SS	95% Ar, 5% CO_2_	Fe	33.89
		Cr	13.11
		Mn	12.36
		Ni	3.76
		Si	2.27
		Cr(VI)	1600 ppm
SMAW‐SS	–	K	26.01
		F	20
		Si	8.32
		Fe	5.39
		Mn	4.83
		Cr	4.71
		Na	2.27
		Ti	1.93
		Al	1.18
		Cr(VI)	38 000 ppm

**Table 2:** Parts per million (ppm) of soluble metals present in the solubilized fraction of welding fume samples as determined using ICP‐OES

Welding fume	Fe (ppm)	Mn (ppm)	Ni (ppm)	Cr (ppm)
GMAW‐MS	0.059	0.643	<0.019	0.016
GMAW‐SS	<0.035	2.347	0.356	0.271
SMAW‐SS	0.06	4.96	0.505	38.059

**Table 3:** Size distribution profile of welding fume particles

	GMAW‐MS	GMAW‐SS	SMAW‐SS
Mean (nm)	108 ± 10.1	150.5 ± 7.1	204.1 ± 2.1
Mode (nm)	58.9 ± 23.5	114.0 ± 16.9	195.1 ± 19.4
Particles/ml	1.4 × 10^8^ ± 2.77 × 10^7^	7.09 × 10^7^ ± 5.57 × 10^6^	1.39 × 10^8^ ± 5.93 × 10^6^
D_50_ (nm)	96.1 ± 9.0	143.2 ± 18.7	193.4 ± 3.8

Note

Average hydrodynamic particle size ± standard error, along with mode, concentration, and distribution of particles according to size is shown. D_50_ indicates the percentage of particles that are below the 50th percentile, which gives an indication of the distribution of particle sizes within the sample

### 3.2 Internalization of welding fume particles by HTR‐8/SVneo cells

**Figure 2:**
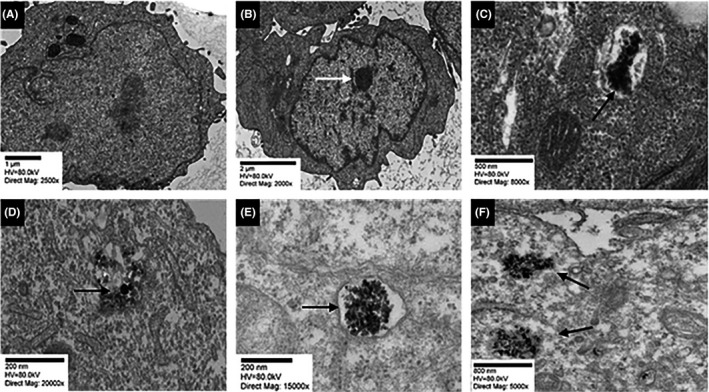
Internalization of welding fume particles by HTR‐8/SVneo cells. Representative images using transmission electron microscopy (TEM) are shown. All treatments were for 24 h. (A) RPMI (negative control), (B) Cr(VI) (positive control; 2000× directing magnification), (C) Cr(VI) (8000× direct magnification), (D) GMAW‐MS, (E) GMAW‐SS, and (F) SMAW‐SS

HTR‐8/SVneo cells exposed to RPMI culture medium for 24 hours (negative controls) contained an intact nucleus and nucleolus, and no cellular abnormalities were observed (Figure 2A). In cells treated with Cr(VI) for 24 hours (positive control), there appeared to be chromosomal condensation (depicted with white arrow; Figure 2B), and the accumulation of Cr particles within the cell was observed (depicted with black arrow, Figure 2C). In Figure 2D‐F, representative images of welding fume particle accumulation in cells after a 24 hours exposure to either MS or SS are shown, though it is more prominent in cells treated with GMAW‐SS and SMAW‐SS.

### 3.3 Negligible endotoxin units (EU) detected

All three welding fume samples were tested for the presence of endotoxin. The average results for GMAW‐MS (0.24 EU/mL), GMAW‐SS (0.17 EU/mL), and SMAW‐SS (0.19 EU/mL) were not statistically significant when compared to the negative control (data not shown).

### 3.4 GMAW‐SS and SMAW‐SS have the greatest effect on cellular viability

**Figure 3:**
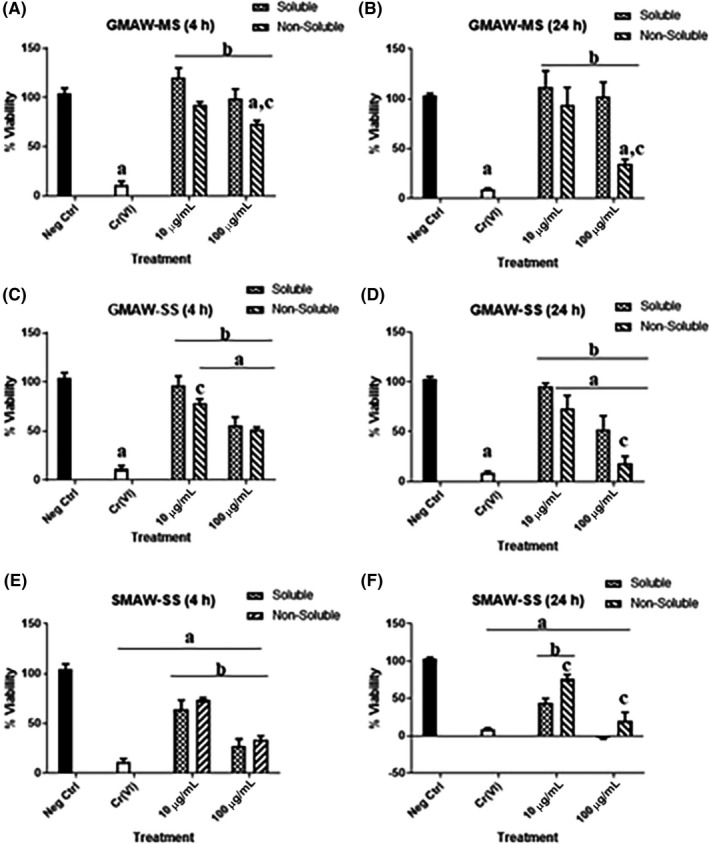
Effects of welding fume treatment on cellular viability. (A‐B) Significant decreases in cellular viability were observed in GMAW‐MS–treated cells at both 4 h and 24 h when exposed to 100 µg/mL of the non‐soluble fraction of welding fume. (C and D) Significant decreases in viability were observed in cells treated with GMAW‐SS at both 4 h and 24 h when compared to controls. (E‐F) SMAW‐SS had the greatest effect on cellular viability compared to GMAW‐MS and SMAW‐SS. ^a^Treatment vs negative Ctrl; ^b^Treatment vs Cr(VI); ^c^Soluble vs non‐soluble (within treatment group)

A significant decrease in the viability of cells treated with the non‐soluble fraction of GMAW‐MS, at both 10 and 100 µg/mL was observed at both 4 and 24 hours (Figure 3A,B, respectively). For cells treated with GMAW‐SS for 4 hours, the non‐soluble fraction of welding fume at 10 µg/mL caused a significant decrease in viability when compared to the soluble fraction. In addition, cells treated with both the soluble and non‐soluble fraction at 100 µg/mL had decreased viability when compared to controls (Figure 3C). At 24 hours, it was only in the 100 µg/mL treatment group that a significant difference in viability was observed when comparing the soluble and non‐soluble fraction‐treated cells (Figure 3D). Finally, for cells treated with SMAW‐SS at both the 4‐ and 24‐hour time points (Figure 3E,F, respectively) cells treated with both 10 and 100 µg/mL displayed significant decreases in viability when compared to controls. Since the dose of 100 µg/mL had such a negative effect on viability, a lower dose of 50 µg/mL was used in all other assays, except for EPR.

### 3.5 Production of ^•^OH is directly proportional to Cr(VI) content in welding fumes

**Figure 4:**
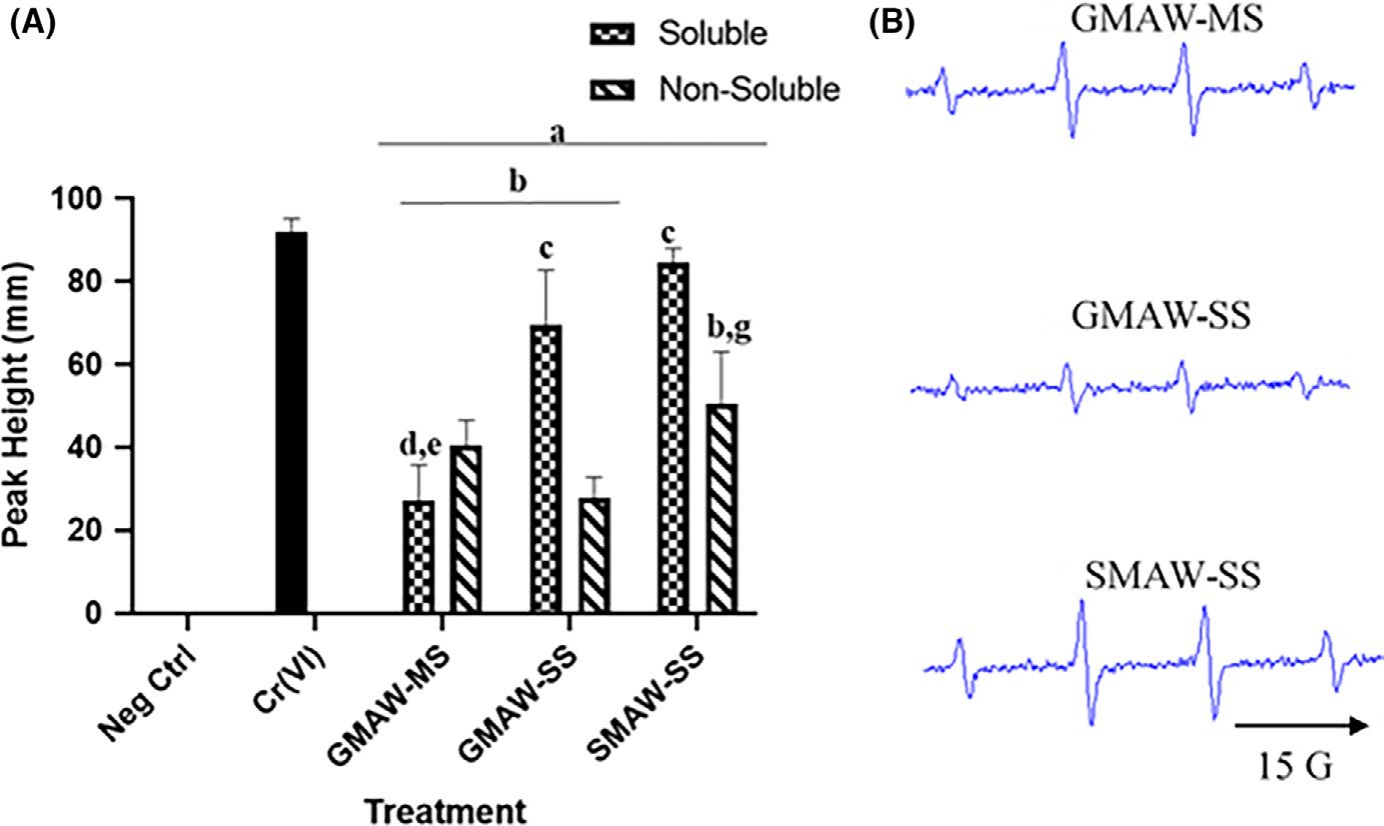
Solubilized welding fumes generate the most ^•^OH. Signal intensity (peak height) was measured and used to determine the relative amounts of ^•^OH produced. (A) GMAW‐MS, GMAW‐SS, and SMAW‐SS exposures in HTR‐8/SVneo cells led to the production of ^•^OH. The soluble fractions of both SS welding fumes generated the greatest production. (B) Representative spectra are shown. ^a^Treatment vs negative control; ^b^Treatment vs Cr(VI); ^c^Soluble vs non‐soluble (within treatment group); ^d^GMAW‐MS vs GMAW‐SS (soluble fraction); ^e^GMAW‐MS vs SMAW‐SS (soluble fraction); ^g^GMAW‐SS vs SMAW‐SS (soluble fraction)

Figure 4A shows the production of ^•^OH in welding fume‐treated trophoblast cells. Though all treatment groups produced significantly more ^•^OH when compared to negative controls, cells treated with SMAW‐SS produced the most ^•^OH, at levels comparable to the Cr(VI)‐positive control. Figure 4B shows representative EPR spectra.

### 3.6 Significant production of intracellular ROS caused by welding fumes

**Figure 5:**
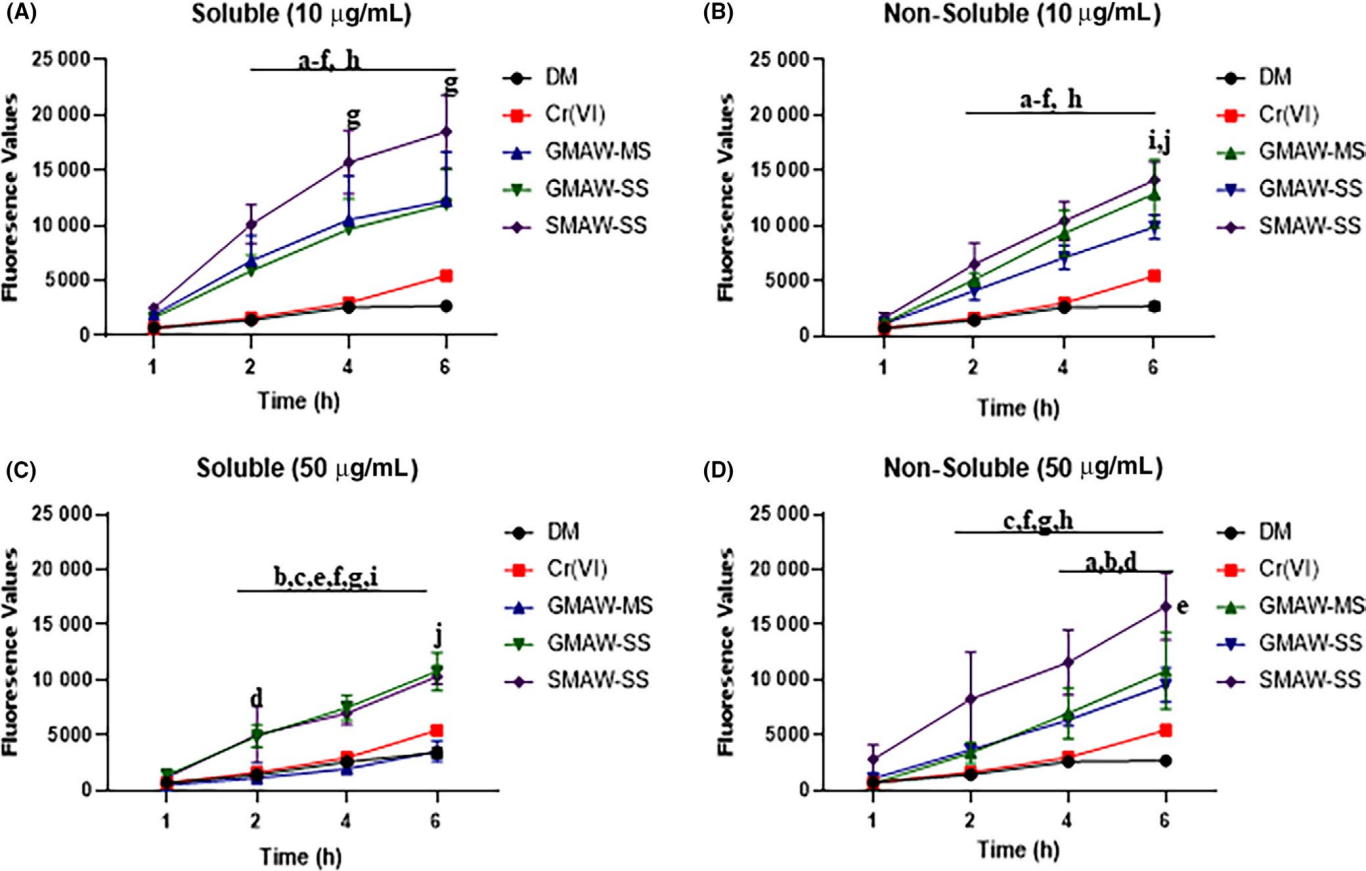
Exposure to SMAW‐SS leads to significant intracellular ROS production in placental cells. (A, B) Both the soluble and non‐soluble fraction (respectively) of SMAW‐SS produced the greatest amounts of intracellular ROS when compared to DM controls. (C) Upon exposure to 50 µg/ml of welding fume, both GMAW‐SS and SMAW‐SS produced significantly more intracellular ROS than GMAW‐MS over the course of time. (D) Compared to negative controls, SMAW‐SS produced the greatest amount of intracellular ROS over time. ^a^GMAW‐MS vs DM; ^b^GMAW‐SS vs DM; ^c^SMAW‐SS vs DM; ^d^GMAW‐MS vs Cr(VI); ^e^GMAW‐SS vs Cr(VI); ^f^SMAW‐SS vs Cr(VI); ^g^SMAW‐SS vs GMAW‐MS; ^h^SMAW‐SS vs GMAW‐SS; ^i^DM vs Cr(VI); ^j^GMAW‐MS vs GMAW‐SS

Over the course of a 6‐h time period, cells exposed to 10 µg/mL of solubilized SMAW‐SS produced significantly greater amounts of intracellular ROS compared with cells exposed to GMAW‐MS and GMAW‐SS (Figure 5A). When cells were exposed to the same dose of welding fume, but the non‐soluble fraction, the production of intracellular ROS in SMAW‐SS treated cells was significantly greater at the 2‐, 4‐, and 6‐hour timepoint when compared to GMAW‐SS, but not GMAW‐MS. A significant difference in intracellular ROS production between GMAW‐MS and GMAW‐SS was only detected at the 6‐hour timepoint (Figure 5B). When the dose of welding fume exposure was increased to 50 µg/mL, cells treated with solubilized GMAW‐SS and SMAW‐SS produced the greatest amounts of intracellular ROS when compared to negative controls (Figure 5C). Finally, when cells were exposed to the non‐soluble fraction of welding fumes at a dose of 50 µg/mL, SMAW‐SS produced significantly greater ROS compared with GMAW‐MS and GMAW‐SS between 2 and 6 hours (Figure 5D).

### 3.7 Exposure to GMAW‐MS causes the greatest increase in IL‐8 production

**Figure 6:**
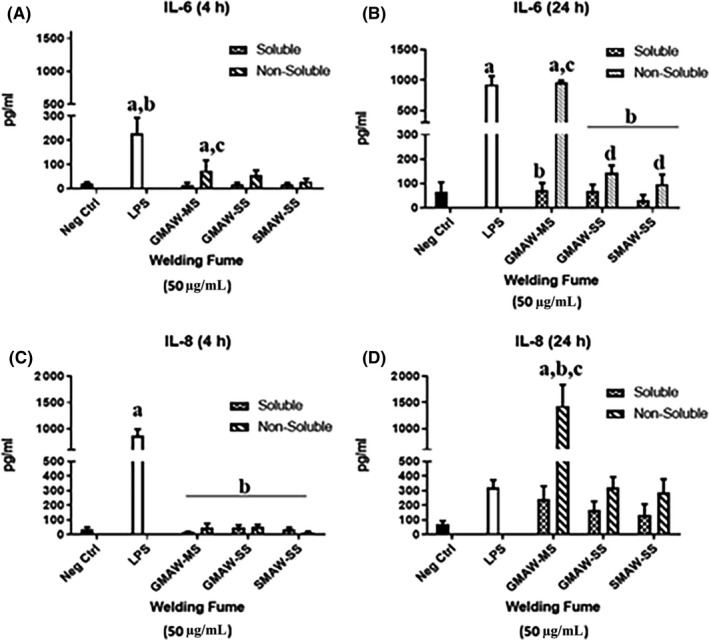
Significant production of IL‐6 and IL‐8 was observed in cells exposed to GMAW‐MS. (A) At 4 h, only the soluble fraction of GMAW‐MS produced significant amounts of IL‐6 when compared to negative controls. (B) At 24 h, a 13‐fold increase in IL‐6 was observed in cells exposed to the soluble fraction of GMAW‐MS when compared to 4 h. (C) At 4 h, no significant increases in IL‐8 production were observed when compared to negative controls. (D) At 24 h, there was a 28‐fold increase in IL‐8 production in cells exposed to GMAW‐MS when compared to 4 h. ^a^Treatment vs negative Control; ^b^Treatment vs LPS; ^c^Soluble vs non‐soluble (within welding fume); ^d^Treatment vs GMAW‐MS (non‐soluble)

Only when cells were exposed to the non‐soluble fraction of GMAW‐MS for 4 hours, there was a significant increase in IL‐6 production when compared to negative controls (Figure 6A). By 24 hours, there was a 13‐fold increase in IL‐6 production (Figure 6B). IL‐8 production was not significantly increased upon exposure to welding fumes at 4 hours (Figure 6C), but at 24 hours there was a 28‐fold increase in cytokine accumulation in cells treated with the non‐soluble fraction of GMAW‐MS (Figure 6D). The detection of IL‐1β and TNFα in supernatants upon exposure to both MS and SS was not significant when compared to controls at either timepoint (data not shown).

### 3.8 Invasive capability of trophoblast cells is impaired when exposed to welding fumes

**Figure 7:**
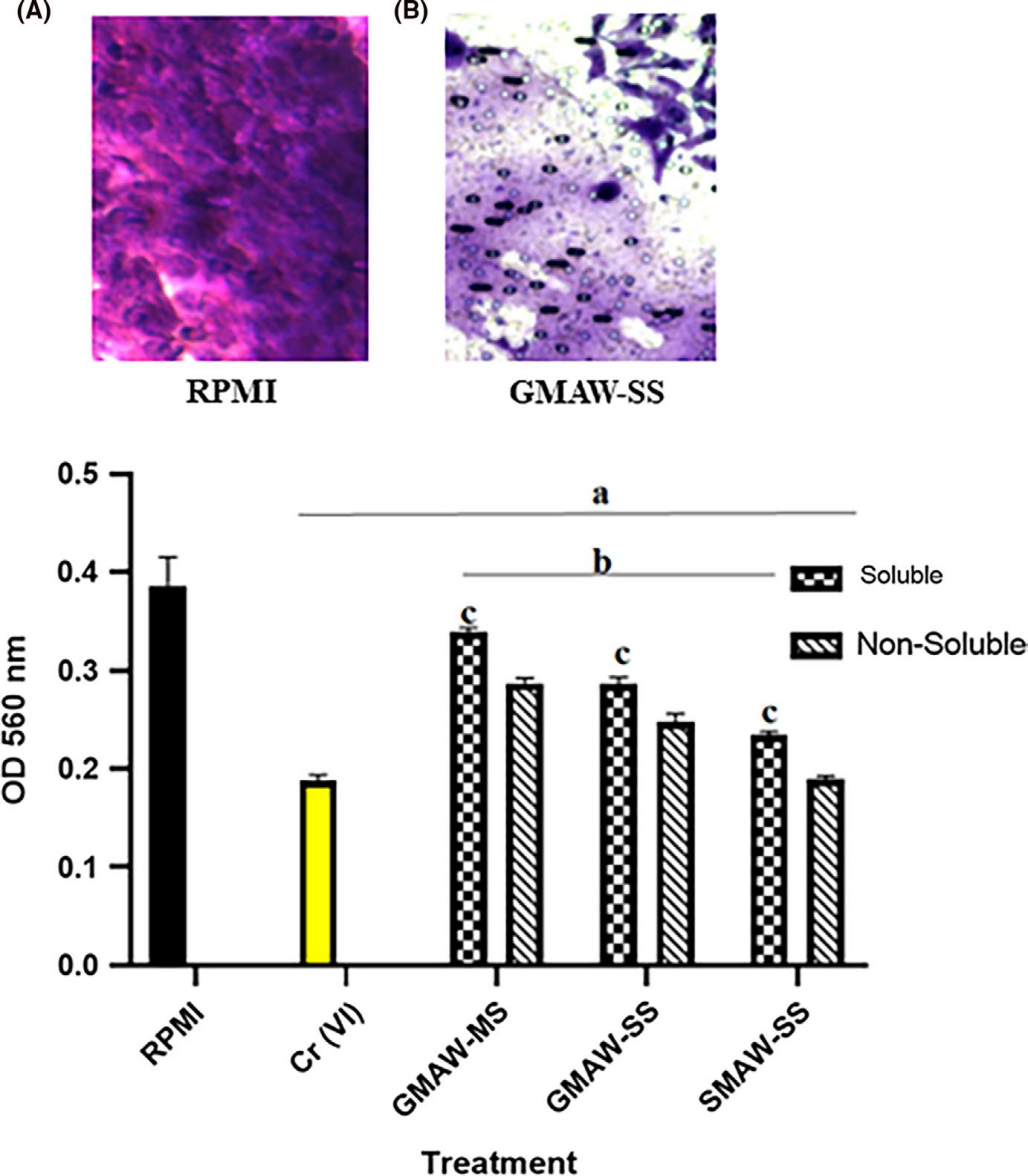
Invasive capability of trophoblast cells decreases upon exposure to welding fumes. (A, B) Representative images of negative control cells and those exposed to welding fume. (C) Compared to RPMI exposed negative controls, cells exposed to welding fumes exhibited significant decreases in their invasive capabilities. Within treatment groups, invasion was significantly decreased in the non‐soluble fraction when compared to the soluble fraction. ^a^Treatment vs RPMI negative control; ^b^Treatment vs Cr(VI); ^c^Soluble vs non‐soluble (within welding fume)

Figure 7A (negative control) and Figure 7B (GMAW‐SS) show representative images of cells that were able to invade through the extracellular matrix. Invasion of cells was also measured by using an extraction buffer on cells and measuring the absorbance at 560 nm. Compared with RPMI controls, all three welding fume types led to significant decreases in the ability of trophoblast cells to invade through the extracellular matrix. This was most notable in SMAW‐SS–treated cells and the non‐soluble fraction of all welding fumes regardless of fume type (Figure 7C).

## 4 DISCUSSION

The effects of welding fume exposure are well characterized in various organ systems, and the International Agency for Cancer Research recently re‐classified welding fumes as Group 1 carcinogens.^27^ However, understanding how welding fume exposures can affect the female reproductive system, specifically the placenta, requires further investigation. With the recent increases in the number of women pursuing careers in skilled trades, it is necessary to identify the potential hazards associated with MS and SS exposure during pregnancy.

We examined the effects of three commonly used welding fumes (GMAW‐MS, GMAW‐SS, and SMAW‐SS) on extravillous trophoblast cells derived from the first trimester. Elemental analysis revealed that GMAW‐MS and GMAW‐SS were composed primarily of Fe and Mn, though GMAW‐SS also contained Cr and Ni. The welding process used to obtain SMAW‐SS did not require a shielding gas to protect the weld area from oxygen and water vapor, which accounts for the high percentage of potassium, used as a coating to protect the electrode. Compared to GMAW‐SS, SMAW‐SS contained almost 24 times the amount of Cr(VI), a known human carcinogen associated with lung, nasal, and sinus cancers.^28^, ^29^, ^30^ Using ICP‐OES, it was determined that Cr(VI) was also the main soluble metal, with Fe, Mn, and Ni being present in significantly lower amounts.

Cr(VI)‐containing compounds are used in many industries and have many applications such as anticorrosion coatings, wood preservation, SS welding, and pigmentation in paint and art supplies.^31^ Each year, it is estimated that more than 500 000 workers in the United States are exposed to these Cr(VI)‐containing compounds through inhalation, and more than 1 million workers are estimated to be exposed through dermal contact.^31^, ^32^ Cr(VI) readily passes through the cellular membrane via anionic transporters, where it is reduced to Cr(III) primarily by ascorbate, although this reduction can also occur by interaction with small thiols such as glutathione, and, to a lesser extent, cysteine. During this reduction, large amounts of free radicals are generated, and Cr(III) can directly bind with DNA, forming Cr‐DNA adducts, thus leading to DNA strand breaks and mutations.^33^, ^34^, ^35^ This likely explains the significant decreases in cellular viability and invasive capabilities observed in trophoblasts exposed to SMAW‐SS, along with the greatest production of ^•^OH and intracellular ROS production when compared to the other two welding fumes. The noticeable decrease in ^•^OH production and intracellular ROS generation in cells exposed to GMAW‐SS may be a direct reflection of the smaller amount of Cr(VI) found in this welding fume. It is unlikely that Cr, however, was the sole contributor toward the production of ^•^OH. Both Fe and Mn, which are present in all three welding fume samples, are known contributors of oxidative stress. In a study conducted by McCarrick et al,^36^ in which they also studied welding fume cytotoxicity, a direct correlation between ROS production and Mn (*R* ^2^ = .92) was observed. Fe is also well known for reacting with hydrogen peroxide, which generates ^•^OH via the Fenton reaction. Badding et al^37^ has previously shown that hydrogen peroxide, produced endogenously by cells when exposed to welding fumes, also leads to the production of ^•^OH, as detected by EPR. In early gestation, it is essential that trophoblast cells invade the maternal decidua, where they remodel spiral arteries from high‐pressure vessels to static high‐capacitance, low pressure vessels by the 10‐12th week of gestational age.^38^ A highly regulated process, abnormalities in trophoblast invasion and proliferation are associated with pathologically deep implantations, such as placental accreta, increta, and percreta, and trophoblast malignancies such as choriocarcinoma.^38^ In patients with preeclampsia, abnormal trophoblast invasion and vascular remodeling are believed to be the cause of oxidative stress to both the trophoblast and developing placenta, leading to fetal growth restriction.^39^, ^40^

During pregnancy, the role of the placenta is multi‐functional. It serves as a source of nutrient and gas exchange at the maternal‐fetal interface, behaves as an immune barrier that protects the developing semi‐allogenic fetus from maternal antibodies, and is also an important endocrine organ that produces many hormones and growth factors.^41^ In women exposed to Cr at the worksite, epidemiological data have shown that these workers have elevated levels of Cr in their blood, urine, umbilical cord, and placental tissue. Adverse reproductive outcomes as a result of these exposures includes spontaneous abortions, fetal birth defects, stillbirths, preterm birth, problems with early emotional development, and neonatal death.^42^, ^43^, ^44^, ^45^, ^46^, ^47^ In studies done on pregnant Sprague Dawley rats, Banu et al^48^ found that exposure to Cr(VI) through drinking water led to increased apoptosis in trophoblast cells, as well in the epithelium of the mesometrial triangle vessels and of the yolk sac epithelium *via* the induction of caspase‐3‐ and p53‐dependent pathways.

While Cr(VI) exposure may have been the driving force behind the toxicity observed in our studies when cells were exposed to GMAW‐SS or SMAW‐SS, welders are exposed to a combination of heavy metals in the workplace. In trophoblasts exposed to GMAW‐MS, which is primarily composed of Fe and Mn, significant decreases in cellular viability and invasion capabilities at 24 hours when compared to controls, along with the significant production of ^•^OH and intracellular ROS, was still observed. In addition, out of the three welding fumes used in our experiments, it was only the GMAW‐MS (non‐soluble fraction) exposed trophoblasts that exhibited production of the pro‐inflammatory cytokines IL‐6 and IL‐8.

Based on the D_50_ data obtained from the particle sizing analysis used in our study, it is likely that many of the particles present in the non‐soluble fraction of all three welding fumes were <100 nm in size and can thus be classified as nanoparticles (having one or more external dimension in the size range of 1‐100 nm).^49^ Several studies have already shown that maternal exposure to ultrafine particles in ambient air pollution is associated with adverse reproductive outcomes such as being born preterm, being small for gestational age, and fetal demise.^50^, ^51^, ^52^ Fewer studies exist on the association between occupational exposure to particles and adverse pregnancy outcomes, despite the fact that pollutant concentration in the workplace can be significantly higher than that of the outdoor environment.^53^

Nanoparticles can directly affect fetal development by crossing the placental barrier and inducing oxidative stress and inflammation in the fetus.^54^, ^55^ They can also indirectly harm the developing fetus as a result of maternal inflammation caused by nanoparticle exposure.^56^, ^57^, ^58^, ^59^ Norlén et al^53^ recently published a prospective study that studied children born in Sweden between 1994 and 2012, whose mothers were occupationally exposed to inorganic particles such as iron dust or fumes as a result of welding, smelting, grinding, or other processing of steel and other materials containing iron. They found, that in mothers who did not have a high absence from work, and who were also exposed to iron‐containing particles throughout their gestation, there were statistically significant increased risks for babies being born small for gestational age, low birth weight, or preterm. A recent longitudinal study conducted by Manangama et al^60^ was also able to link maternal occupational exposures to nanoparticles to babies being born small for gestational age but made no mention of welders.

During pregnancy, the placenta plays a very active role in iron transport to the fetus. As pregnancy progresses, demand for iron increases, since it is an essential component of many enzymes and hemoproteins needed for normal cellular function, as well as proper placental development.^61^ In fact, it is common for infants born to anemic women to have normal hemoglobin levels, as it is now widely accepted that the fetus is able to adequately obtain iron despite maternal deficiency, and is the driving force behind iron transfer across the placenta.^61^, ^62^ The fact that the non‐soluble fraction of GMAW‐MS was the only welding fume to elicit a cytokine response in trophoblasts could then potentially be explained by the predominant presence of Fe (54.76%). In women that develop gestational diabetes, the most common metabolic disorder during pregnancy that favors a pro‐inflammatory state, it has been found that iron overload and iron metabolizing disorders are often associated with glucose‐related imbalances.^63^, ^64^, ^65^, ^66^ Further studies are necessary to fully understand why only GMAW‐MS–exposed cells had significant increases in IL‐6 and IL‐8, since GMAW‐SS also contained Fe, but did not cause increased cytokine production.

In 2007, Antonini and Roberts published a report,^67^ in which rats were exposed to both welding fumes and *Listeria monocytogenes*, since welders tend to have increased reports of pulmonary infections compared to the general population. They found, that in animals pretreated with Cr (a major component of SS), pulmonary cytokine expression in bacteria‐exposed rats was suppressed. They concluded that Cr was most likely acting as an immune suppressant in these animals. Though our trophoblasts were not exposed to bacteria, GMAW‐SS and SMAW‐SS both contain Cr, further leading us to believe that Fe could be the primary cause for cytokine production in cells.

The results of our study show that GMAW‐MS, GMAW‐SS, and SMAW‐SS are all cytotoxic to trophoblasts. While GMAW‐MS was the only welding fume to cause increased IL‐6 and IL‐8 production, all three welding fume exposures led to increased production of ^•^OH, increased production of intracellular ROS, and decreased invasion capabilities. As more women are turning to skilled trades such as welding, identifying how these occupational exposures could impact maternal and fetal health is necessary. Understanding how the varying combinations of heavy metals and other materials present in MS and SS welding fumes, along with fume particle size, adversely affects the maternal‐fetal interface and gestational outcomes should be considered an emerging issue.

## CONFLICT OF INTEREST

The authors of this manuscript do not have any conflicts of interest, financial, or otherwise, to declare.

## DISCLAIMER

The findings and conclusions in this report are those of the authors and do not necessarily represent the official position of the National Institute for Occupational Safety and Health, Centers for Disease Control and Prevention. The mention of brand name does not constitute product endorsement.
